# Improved Social Functioning Mediated the Effects of Adolescents' Sports Participation on Self‐Harm and Psychological Wellbeing: A Prospective Cohort Study

**DOI:** 10.1111/sltb.70141

**Published:** 2026-10-07

**Authors:** Mark Lawrence Wong

**Affiliations:** ^1^ Department of Social and Behavioural Sciences City University of Hong Kong Hong Kong Hong Kong

**Keywords:** good health and well‐being, health promotion, mortality, physical activities, self‐injury behaviors, suicidal ideation

## Abstract

**Introduction:**

The prevalence of self‐harm, regardless of the presence of suicidal intent or not, has been increasing, and effective and accessible intervention strategies are needed. This study investigated whether sports participation in adolescence serves as a protective factor against the future development of self‐harm or poor psychological wellbeing, as well as the relevant psychosocial mechanisms.

**Methods:**

A secondary data analysis was conducted using a nationally representative cohort in the United Kingdom, in which participants (*n* = 7361, 55.4% female) completed measures of sports participation (age 17), the number of close friends (age 19), life satisfaction (age 20), and self‐harm and poor psychological wellbeing (age 25).

**Results:**

More frequent sports participation was prospectively associated with a lower risk of self‐harm and better psychological wellbeing. Path analyses supported a significant serial mediation model whereby the effects of sports participation on self‐harm and psychological wellbeing are mediated by an increased number of close friends and higher levels of life satisfaction.

**Conclusion:**

These findings indicate that sports participation has potential value in optimizing mental health, particularly in reducing the likelihood of self‐harm and improving psychological wellbeing. In addition, improved social relationships and life satisfaction mediated the impact of sports participation on self‐harm and psychological wellbeing. Overall, the findings lay a solid foundation for further testing the benefit of encouraging sports participation among adolescents to optimize interpersonal functioning and mental health outcomes.

## 1 Introduction

Self‐harm is defined as a self‐inflicted physical injury regardless of suicidal intent (World Health Organization. Regional Office for the Eastern Mediterranean 2019). Self‐harm has consistently been found to correlate with poor psychological wellbeing, increased mortality, hospitalization, mental illness and other adverse consequences (Beckman et al. 2016; Olfson et al. 2018; Tepman and Wong 2024). Moreover, the prevalence of self‐harm among adolescents and young adults has shown a steady upward trend (Griffin et al. 2018; Trafford et al. 2023), tendering strategies to prevent self‐harm and enhance psychological wellbeing a clinical and public health priority. Accordingly, this paper aims to investigate the effects and underlying mechanisms of sports participation on self‐harm and psychological wellbeing, given that sports, as a lifestyle intervention, has the potential to be accessible to young people and relevant key stakeholders without associated social stigma.

### 1.1 Sports, Self‐Harm and Psychological Wellbeing

Recent findings demonstrated mixed results regarding the associations between sports participation and indicators of mental health, including depressive mood, self‐esteem, and perceived social support (Boone and Brausch 2016; Latina et al. 2022). For example, findings from a community study of youth (age 14–25) showed that more frequent engagement in physical exercise was correlated with reduced self‐harm, an association that remained independent of individual depression symptom severity (Boone and Brausch 2016). Among young adults with a history of self‐harm, engaging in sport or physical exercise was rated as a helpful strategy for resisting self‐injurious urges. Conversely, other studies have found that exercise or physical activity was not correlated with suicidality or psychological wellbeing (Huang et al. 2024; Lee et al. 2025).

One possible reason for this inconsistency is the marked heterogeneity in the measurement of sports participation (Huo et al. 2024). Specifically, some studies have operationalized physical activity using binary measures (i.e., presence versus absence of sport or exercise engagement) (e.g., Plener et al. 2013), whereas others measured the frequency of participation at specific intensity levels (e.g., Huang et al. 2024). In addition, a meta‐analysis suggested that despite an increasing number of studies assessing the cross‐sectional relationship between physical exercise and self‐harm or other mental health indicators among adolescents, few longitudinal studies have clarified their directional relationship (Lan et al. 2020). For instance, in a population‐based co‐twin control sample (*n* = 18,234), parent‐reported sports participation at age 15 prospectively predicted a lower likelihood of self‐harm at age 18 (O'Reilly et al. 2022). Similarly, a recent longitudinal study of 998 adolescents (mean age = 13.9 years) found that sports participation prospectively reduced self‐harm one year later (Latina et al. 2022). The authors additionally found that this association was mediated by self‐esteem, although self‐esteem and self‐harm were measured at the same time‐points, precluding inference regarding their directional relationship. Accordingly, the prospective association between sports participation and self‐harm, and the underlying mechanisms require further investigation.

The effects of sports participation on self‐harm and psychological wellbeing may be explained by enhanced social relationships and greater life satisfaction derived from participation. Evidence from cross‐sectional studies indicates that adolescents participating in sports generally report lower rates of self‐harm and suicidality, alongside better psychological wellbeing. Furthermore, these associations are mediated by enhanced social support, stronger peer relationships, and a higher sense of life satisfaction (Babiss and Gangwisch 2009; Taliaferro et al. 2010). Enhanced social support and peer relationships can be fostered when adolescents interact with their peers, team‐mates, and coaches. Cognitive‐behavioral theories also support the premise that stronger social relationships serve as vital coping resources against stressful life events (Rudd 2000). Moreover, according to the Interpersonal Theory of Suicide, sports participation and improved social relationships can be conceptualized as important factors that foster a sense of belonging within a broader community. A sports team or a friend group, for example, subsequently acts as a protective factor against suicidality and self‐harm (Barzilay et al. 2015). Enhanced social relationships and a stronger sense of belonging are also consistent predictors of better psychological wellbeing (Arslan 2018). While theoretical perspectives suggest that improved interpersonal interactions represent a primary psychosocial mechanism underlying the benefits of sports participation, empirical data from longitudinal studies remain necessary to test and confirm these dynamic associations (Latina et al. 2022).

### 1.2 Current Study

The primary aim of this study was to investigate the longitudinal effects and the underlying psychosocial mechanisms linking adolescents' sports participation to self‐harm and psychological wellbeing in young adulthood. Importantly, self‐harm in the present study was operationalized broadly, capturing self‐injurious behaviors regardless of the presence or absence of suicidal intent. In the context of self‐harm prevention, investigating sports participation holds significant public health value because: (1) adolescents generally hold positive perceptions of sport given its physical and mental health benefits; and (2) sports participation can be readily integrated into existing school‐based and public health promotion campaigns. Understanding these psychosocial mechanisms can help identify the individuals most suitable for sports‐focused self‐harm prevention programs. Building on the results of existing cross‐sectional and longitudinal literature, the current study measured the predictor (sports participation), mediators (social relationships and life satisfaction), and outcomes (self‐harm and psychological wellbeing) across four different time points, thereby enabling robust inference regarding their temporal associations. Also, the use of a nationally representative cohort from the United Kingdom enhances the generalizability of the findings. It was hypothesized that sports participation would prospectively predict a lower risk of self‐harm (irrespective of intent) and greater psychological wellbeing in young adulthood, and that these protective associations would be mediated by stronger social relationships and higher life satisfaction.

## 2 Methods

### 2.1 Design

To address the research questions regarding sports participation and self‐harm, a secondary data analysis was conducted using a nationally representative prospective cohort from the UK—the Next Steps study (Wu et al. 2024). Participants of the Next Steps were born between 1 September 1989 and 31 August 1990, and were attending English state or independent schools in February 2004. Informed consent was obtained from participants and their parents/guardians prior to data collection, and participants were explicitly informed that they could withdraw from the study anytime. Ethics approval was obtained from the [anonymized] prior to data collection, and the details of the cohort profile are reported elsewhere (Wu et al. 2024).

For the current study, data were extracted across four time points when participants were aged 17 (predictor), 19 (mediator), 20 (mediator), and 25 years (outcome). Specifically, the predictor was the frequency of sports participation at age 17; the mediators were the number of friends at age 19 and overall life satisfaction at age 20; and the primary outcomes were self‐harm behavior and psychological wellbeing at age 25 (see Measures for details).

### 2.2 Participants

In Next Steps, 11,449 cohort members participated at age 17, with 9799 participating at age 19, 8682 at age 20 and 7707 at age 25 years. In this study, participants' data were included and analyzed if individuals had participated across all four time points and provided valid responses to items measuring sports participation and either self‐harm or psychological wellbeing. The final analytical sample included 7361 participants; detailed baseline demographic characteristics and sample descriptive information are reported in Table 1.

**TABLE 1:** Descriptive information of the sample.

	*N*	Mean (SD)/%
Gender		
Female	3497	55.4%
Male	2814	44.6%
Ethnicity		
White (British, Irish and others)	5103	69.3%
Mixed	347	4.7%
Asian	1288	17.5%
Black	569	7.7%
Others	54	0.7%
Household weekly income	6858	£397.83 (3.196)
Body Mass Index	7005	25.24 (.069)
Sports frequency		
Most days	1244	19.7%
> 1/week	1151	18.2%
1/week	1020	16.2%
< 1/week	778	12.3%
Hardly ever	1388	22.2%
Never	728	11.5%
Number of close friends		
None	55	1.0%
1	195	3.4%
2–3	1752	30.4%
4–5	1916	33.1%
6–9	1018	17.6%
> 10	847	14.6%
Life satisfaction		
Very satisfied	1510	27.9%
Fairly satisfied	2828	52.3%
Neither satisfied nor dissatisfied	649	12.0%
Fairly dissatisfied	315	5.8%
Very dissatisfied	109	2.0%
Self‐harm in last year		
Yes	255	3.5%
No	6954	96.5%
GHQ—12 total score	7361	2.30 (.036)

Abbreviation: GHQ‐12, General Health Questionnaire‐12 items.

### 2.3 Measures

For sports participation, participants were asked to indicate how often they do sports like football, aerobics, dance classes, or swimming. Responses were recorded on a 6‐point scale, ranging from 1 (most days) to 6 (never). For number of close friends, participants were asked how many close friends they had, with close friends defined as those with whom they could discuss personal difficulties. Responses were provided on a 6‐point scale ranging from 1 (none) to 6 (10 or more). For life satisfaction, participants indicated how satisfied or dissatisfied they were with how their life had turned out so far, using a 5‐point scale ranging from 1 (very satisfied) to 5 (very dissatisfied). For all items, responses of “don't know” were treated as missing data. Prior to main analyses, measures of sports participation frequency and life satisfaction were reverse‐recoded so that higher values consistently represented greater frequency, higher satisfaction, or more favorable outcomes.

Self‐harm was assessed using a single binary item asking participants whether they had “hurt [themselves] on purpose in any way during the previous year” (Yes/No). This item captures broad, past‐year self‐harm engagement irrespective of suicidal intent; thus encompassing both non‐suicidal self‐injury (NSSI) and suicidal attempts. Psychological wellbeing was evaluated using the 12‐item General Health Questionnaire (GHQ‐12) (Gureje and Obikoya 1990), with participants responding on a 4‐point Likert scale. Total scores ranged from 12 to 48, where higher scores represented higher levels of psychological distress (and lower psychological wellbeing). The GHQ‐12 demonstrated excellent internal consistency in the present sample (*α* = 0.899).

Baseline demographic and socio‐economic characteristics of the participants—including age, gender, ethnicity, body mass index, weekly family income, and educational attainment are presented in Table 1.

### 2.4 Data Analysis Plan

JAMOVI (version 2.4) was used for all statistical analysis (The Jamovi Project 2023). Zero‐order bivariate correlational analyses were conducted to evaluate the associations among the hypothesized predictor, mediators, and outcome variables. To assess missingness mechanisms, Little's Missing Completely at Random test was performed, where a *p*‐value > 0.05 indicates the data were missing completely at random. To test the main hypotheses, path analyses were conducted to examine the direct and indirect effects of sport participation on self‐harm and psychological wellbeing. In the path model, age‐17 sports participation was specified as the predictor, age‐19 number of friends as the primary mediator, age‐20 life satisfaction as the secondary mediator, and age‐25 self‐harm and psychological wellbeing as the primary outcomes.

Diagonally Weighted Least Square (DWLS) estimation was employed to account for non‐normal and categorical/ordinal data. Model fit for the hypothesized serial mediation model was evaluated using standard goodness‐of‐fit criteria: Comparative Fit Index (CFI > 0.95); Root Mean Square Error of Approximation (RMSEA < 0.06 with a *p*‐value > 0.05); and the Standardized Root Mean Square Residual (SRMR < 0.08) (Hu and Bentler 1999). Bootstrapping with 5000 resamples was used to estimate standard errors. A significant indirect mediation effect was inferred if the 95% CI did not contain zero.

## 3 Results

Results from Little's MCAR test indicated that the data were missing completely at random, *χ* ^2^(9) = 9.714, *p* = 0.374. Attrition was also found not to be associated with participants' number of close friends, *F*_1,9614,_=0.046, *p* = 0.831; life satisfaction, *χ* ^2^(4) = 5.897, *p* = 0.207; self‐harm, *χ* ^2^(1) = 0.327, *p* = 0.568; or level of psychological wellbeing, *F*_1,7360_ = 2.394, *p* = 0.122.

Results from Spearman's rank correlation analyses indicated that more frequent sports participation was significantly correlated with a greater number of close friends, *ρ* = 0.204, *p* < 0.001; a higher level of life satisfaction, *ρ* = 0.081, *p* < 0.001; a lower risk of self‐harm, *ρ* = −0.041, *p* < 0.01; and lower psychological distress (higher psychological wellbeing), *ρ* = −0.204, *p* < 0.001. A greater number of close friends and higher levels of life satisfaction were also found to be significantly inter‐correlated, as well as correlated with self‐harm and psychological wellbeing (Table 2).

**TABLE 2:** Zero‐order correlation among core study variables.

	1	2	3	4
1. T1 Sports participation frequency	—			
2. T2 Number of Close Friends	0.204***	—		
3. T3 Life Satisfaction	0.081***	0.105***	—	
4. T4 Self‐harm	−0.041**	−0.056***	−0.080***	—
5. T4 GHQ‐12	−0.087***	−0.061***	−0.172***	−0.206***

Abbreviation: GHQ‐12: General Health Questionnaire‐12 items.

** *p* < 0.01.

*** *p* < 0.001.

### 3.1 Prospective Analyses on Sports Participation, Number of Close Friends, Life Satisfaction, Self‐Harm and Psychological Wellbeing

Results from the path analysis (Figure 1) demonstrated excellent model fit to the data, CFI = 0.983, RMSEA = 0.052, *p* = 0.380, SRMR = 0.009. Self‐harm was directly predicted by a lower number of close friends, beta = −0.043, *p* = 0.004, and a lower level of life satisfaction, beta = −0.092, *p* < 0.001, but not significantly by sports participation frequency, beta = −0.015, *p* = 0.325. For indirect effects, more frequent sports participation indirectly predicted a lower likelihood of self‐harm, via (1) an increased number of close friends, beta = −0.007, 95% CI: −0.001 to −0.0001, *p* = 0.006; (2) greater life satisfaction, beta = −0.010, 95% CI: −0.002 to −0.001, *p* < 0.001; and (3) a chained mediation pathway through both an increased number of close friends and subsequent greater life satisfaction, beta = −0.002, 95% CI: −0.0001 to −0.00001, *p* < 0.001 (Table 3).

**FIGURE 1:**
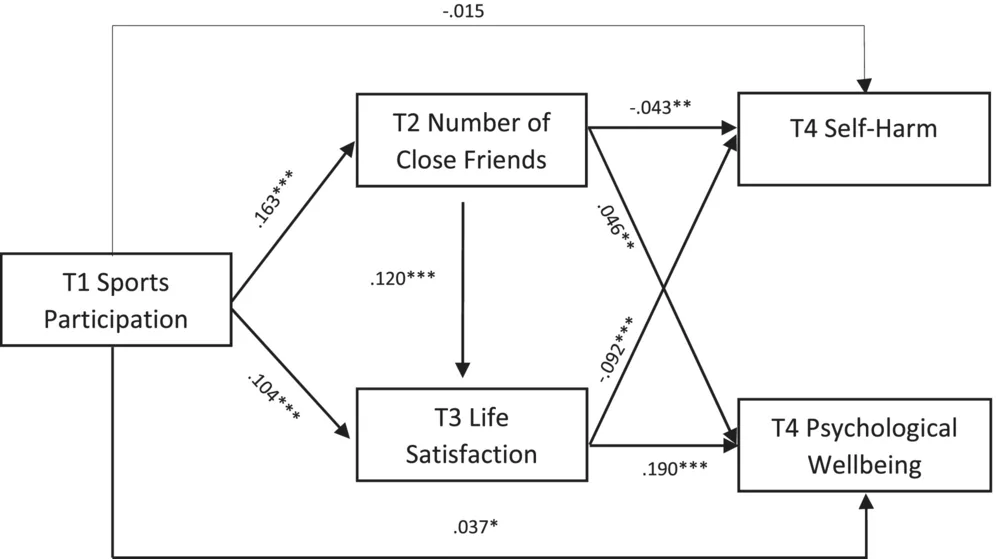
Path analyses of the effects of sports participation on self‐harm and psychological wellbeing. Model Fit Indices: CFI = 0.983. RMSEA = 0.052, 95% CI = 0.031–0.077, *p* = 0.0379, SRMR = 0.009. *p < 0.05, **p < 0.01, ***p < 0.001.

**TABLE 3:** Effects of sports participation on self‐harm and psychological wellbeing.

	T4 self‐harm			T4 psychological wellbeing		
	OR	Confidence interval		B	Confidence interval	
		2.5%	97.5%		2.5%	97.5%
Direct effects						
T1 Sports Participation	0.998	−0.005	0.002	−0.067*	−0.014	−0.119
T2 Number of Close Friends	0.993**	0.002	0.012	−0.123**	−0.200	−0.050
T3 Life Satisfaction	1.019***	−0.026	−0.012	−0.643***	0.540	0.745
Indirect effects						
Sports Participation → Number of Close Friends	0.999**	−0.001	−0.0001	−0.013**	−0.005	−0.022
Sports Participation → Number of Close Friends → Life Satisfaction	0.999***	−0.0001	−0.00001	−0.007***	−0.005	−0.009
Sports Participation → Life Satisfaction	0.999***	−0.002	−0.001	−0.035***	−0.024	−0.048
Number of Close Friends → Life Satisfaction	0.998***	0.001	0.003	−0.062***	−0.081	−0.044

Abbreviations: B, unstandardized regression coefficient; OR, odd ratios.

* *p* < 0.05.

** *p* < 0.01.

*** *p* < 0.001.

Greater psychological wellbeing (lower psychological distress) was directly predicted by more frequent sports participation, beta = −0.037, *p* = 0.038; a higher number of close friends, beta = −0.046, *p* = 0.005; and a higher level of life satisfaction, beta = −0.190, *p* < 0.001 (Figure 1). In addition, more frequent sports participation indirectly predicted greater psychological wellbeing via (1) an increased number of close friends, beta = −0.013, 95% CI: −0.022 to −0.005, *p* = 0.002; (2) greater life satisfaction, beta = −0.035, 95% CI: −0.048 to −0.024, *p* < 0.001; and (3) a chained mediation pathway of increased number of close friends and subsequently greater life satisfaction, beta = −0.007, 95% CI: −0.005 to −0.009, *p* < 0.001 (Table 3).

## 4 Discussion

In this study, the primary research question addressed the longitudinal effects and psychosocial mechanisms of adolescents' sports participation on the subsequent development of self‐harm (conceptualized broadly, irrespective of suicidal intent) and poor psychological wellbeing. The results indicated that more frequent sports participation prospectively predicted a reduced risk of self‐harm and better psychological wellbeing. A greater number of close friends and higher life satisfaction further mediated the effects of sports participation on self‐harm and psychological wellbeing.

### 4.1 Effects of Frequent Sports Participation on Self‐Harm and Psychological Wellbeing

While existing cross‐sectional studies have yielded mixed evidence regarding the association between sports participation and self‐harm, our results demonstrated a significant negative association between frequent sports participation and both self‐harm and poor psychological wellbeing. Notably, our findings align with literature across the self‐harm spectrum, encompassing studies that have examined both non‐suicidal self‐injury (NSSI) and the combined self‐harm construct in other regions, such as Austria, Germany, and Switzerland (Plener et al. 2013), Norway (Grasdalsmoen et al. 2020), and the United States (Boone and Brausch 2016; Klonsky and Glenn 2008).

A core strength of the present study lies in its use of a nationally representative UK sample, which enhances the generalizability of the findings. It is worth noting that the 12‐month self‐harm prevalence observed in our sample (3.5%) is conservative compared to population‐based estimates utilizing multi‐behavioral inventories in other UK prospective cohorts, such as the Millennium Cohort Study (24.1%; Patalay and Fitzsimons 2021). This lower baseline prevalence likely reflects the known psychometric characteristics of direct single‐item queries, which inherently yield lower reporting rates than comprehensive checklists (Muehlenkamp et al. 2012; Swannell et al. 2014) as participants often reserve endorsement for higher‐severity, less ambiguous presentations. Still, this conservative operational threshold reinforces the empirical validity and robustness of our findings. Given that low baseline outcome prevalence can attenuate statistical power in prospective regression models (e.g., King and Zeng 2001), the fact that sports participation and both prospective mediators still significantly predicted self‐harm underscores the important protective signal of these social buffers.

The divergence between our findings and certain previous studies (e.g., Huang et al. 2024; Lee et al. 2025) may stem from differences in how sports participation was operationalized. For instance, specific types (e.g., organized, team versus individual sports) or levels of intensity (Huang et al. 2024; Latina et al. 2022; Lee et al. 2025) were not captured in the present study. Furthermore, sample age profiles varied across studies; the present prospective cohort tracked participants from late adolescence (age 17) to young adulthood (age 25), whereas studies reporting non‐significant associations evaluated broader adult populations (ages 18–70) (Lee et al. 2025) or childhood to early adolescence cohorts (ages 10–14) (Huang et al. 2024). From a developmental perspective, adolescents and young adults face increasing academic, social, and interpersonal demands, as they transition into adulthood, coinciding with a well‐documented decline in sports participation (Kwon et al. 2015; Telama and Yang 2000). The present findings highlight that maintaining sports participation during this vulnerable developmental window plays a protective role in youth mental health.

### 4.2 Psychosocial Mechanisms Underlying the Impact of Sports Participation on Self‐Harm and Psychological Wellbeing

To the best knowledge of the author, this is one of the first longitudinal studies to investigate the psychosocial mechanisms through which adolescent sports participation influences self‐harm and psychological wellbeing. Beyond physiological and cognitive benefits (Lee et al. 2014), our findings demonstrate that sports participation benefits psychosocial aspects in adolescents' lives—specifically, a higher number of close friends and greater life satisfaction—which subsequently predict reduced self‐harm and enhanced psychological wellbeing.

These result patterns align with the Interpersonal Theory of Suicidality (Barzilay et al. 2015). More frequent sports participation likely offers structured opportunities for meaningful peer and mentor interactions, facilitating the formation and maintenance of close friendships and thereby mitigating thwarted belongingness. Concurrently, these findings s could be conceptualized through a cognitive‐behavioral framework (Rudd 2000): an expanded social network provides adaptively coping resources during acute stress, while sports engagement instils a sense of mastery and positive affect. This positive reinforcement may directly counter negative cognition regarding the self, world, and future (Wang et al. 2025). Although the present study focused on self‐harm and psychological wellbeing, the observed prospective gains in social connectedness and life satisfaction suggest that sports participation may yield broader mental health benefits—potentially reducing loneliness, low self‐esteem, and suicidal ideation (Babiss and Gangwisch 2009; Grasdalsmoen et al. 2020; Nicolaisen and Thorsen 2017). Future prospective research should examine whether adolescent sports participation exerts domain‐specific protective effects or operates as a more general buffer across a wider spectrum of mental health outcomes.

### 4.3 Limitations

A key limitation, common to nationally representative prospective cohort studies, is the reliance on self‐reported measures. Specifically, self‐harm was assessed using a single binary item that did not evaluate suicidal intent. Consequently, this measure captures broad self‐harm engagement—encompassing both NSSI and suicide attempts—without differentiating between them. Furthermore, single‐item queries inherently carry psychometric constraints: behavioral checklist inventories systematically yield higher self‐reported self‐injury rates by prompting specific acts (Robinson and Wilson 2020), whereas direct single items typically capture a more conservative, high‐severity threshold due to participant reporting biases.

Also, only the frequency of sports participation was measured, whereas previous studies suggest that sport modality (e.g., organized vs. unorganized) physical activity intensity are important factors influencing mental health outcomes (Latina et al. 2022; Lee et al. 2025). Future studies incorporating objective measures (e.g., accelerometery), informant reports, and detailed activity profiles will be helpful to refine these findings. Furthermore, while the observed prospective associations were statistically significant, the effect sizes were small. However, these effect sizes are comparable to those reported in other longitudinal cohorts investigating sports participation and self‐harm (Latina et al. 2022; O'Reilly et al. 2022). These modest effects likely reflect the complex, multifactorial etiology of self‐harm, wherein sports participation represents one of several contributing buffers. Still, establishing these associations within a nationally representative prospective cohort provides solid evidence that frequent sports participation prospectively predicts lower self‐harm risk and superior psychological wellbeing. Finally, considerable sample attrition occurred across study waves, consistent with similar prospective cohort studies (e.g., O'Reilly et al. 2022). Still, baseline attrition analyses confirmed that participant drop‐put was not significantly associated with self‐harm, baseline psychological wellbeing, number of close friends, or life satisfaction. To build upon these foundational findings, future research should utilize prospective designs with validated multi‐item psychometric batteries that explicitly delineate NSSI from suicidal intent, incorporate objective physical activity metrics, and evaluate qualitative dimensions of sport engagement across diverse youth populations.

## 5 Conclusion

In conclusion, while self‐harm and poor psychological wellbeing have risen steadily among young people in recent years, the present findings indicate that frequent sports participation serves as a protective factor against subsequent adverse mental health outcomes. Beyond physical benefits, sports participation prospectively predicted enhanced social relationships and life satisfaction, which in turn mediated reductions in self‐harm and improvements in psychological wellbeing. Taken together, these findings provide proof‐of‐concept evidence highlighting the promise of school‐ and community‐based sports initiatives as low‐stigma avenues for fostering youth resilience. Of note, these findings highlight the necessity of future longitudinal research incorporating validated, multi‐item psychometric measures to confirm these protective mechanisms and inform evidence‐based youth mental health strategies.

## Author Contributions

**Mark Lawrence Wong:** conceptualization, investigation, writing – original draft, methodology, validation, visualization, writing – review and editing, software, formal analysis, project administration, data curation, resources.

## Funding

The author has nothing to report.

## Ethics Statement

Ethics approval was obtained from the NHS Research Ethics Committee (NRES)—REC Reference 14/LO/0096 before data collection.

## Consent

All participants and their parent/guardian provided informed consent before data collection.

## Conflicts of Interest

The author declares no conflicts of interest.

## Data Availability

The data that support the findings of this study are openly available in UK Data service at https://beta.ukdataservice.ac.uk/datacatalogue/series/series?id=2000030, reference number SN: 2000030.
